# Feasibility and sustainability of an interactive team-based learning method for medical education during a severe faculty shortage in Zimbabwe

**DOI:** 10.1186/1472-6920-14-63

**Published:** 2014-03-28

**Authors:** Jacob Gray, Golden T Fana, Thomas B Campbell, James G Hakim, Margaret Z Borok, Eva M Aagaard

**Affiliations:** 1 Division of Infectious Disease, Department of Medicine, University of Colorado School of Medicine, 12700 E 19th Ave Campus Box B168, Aurora, CO 80045, USA; 2 Department of Medicine, University of Zimbabwe College of Health Sciences, Parirenyatwa Hospital, Avondale, Harare, Zimbabwe; 3 Department of Medicine, University of Colorado School of Medicine, 12631 E 17th Ave Campus Box B178, Aurora, CO 80045, USA; 4 NECTAR MEPI Program, Department of Medicine, College of Health Sciences, University of Zimbabwe, P.O. Box A178, Avondale, Harare, Zimbabwe; 5 Alaska Native Medical Center, 4000 Diplomacy Drive, Anchorage, AK 99508, USA

**Keywords:** Team-based learning, TBL, Resource-limited, Medical education, NECTAR, MEPI, HIV

## Abstract

**Background:**

In 2010, in the midst of the human immunodeficiency virus (HIV) epidemic in Zimbabwe, 69% of faculty positions in the Department of Medicine of the University of Zimbabwe College of Health Sciences (UZ-CHS) were vacant. To address the ongoing need to train highly skilled HIV clinicians with only a limited number of faculty, we developed and implemented a course for final-year medical students focused on HIV care using team-based learning (TBL) methods.

**Methods:**

A competency-based HIV curriculum was developed and delivered to final-year medical students in 10 TBL sessions as part of a 12 week clinical medicine attachment. A questionnaire was administered to the students after completion of the course to assess their perception of TBL and self-perceived knowledge gained in HIV care. Two cohorts of students completed the survey in separate academic years, 2011 and 2012. Descriptive analysis of survey results was performed.

**Results:**

Ninety-six of 120 students (80%) completed surveys. One hundred percent of respondents agreed that TBL was an effective way to learn about HIV and 66% strongly agreed. The majority of respondents agreed that TBL was more stimulating than a lecture course (94%), fostered enthusiasm for the course material (91%), and improved teamwork (96%). Students perceived improvements in knowledge gained across all of the HIV subjects covered, especially in challenging applied clinical topics, such as management of HIV antiretroviral failure (88% with at least a “large improvement”) and HIV-tuberculosis co-infection (80% with at least a “large improvement”).

**Conclusions:**

TBL is feasible as part of medical education in an African setting. TBL is a promising way to teach challenging clinical topics in a stimulating and interactive learning environment in a low-income country setting with a high ratio of students to teachers.

## Background

An economic crisis in Zimbabwe from 1999-2009 resulted in an overwhelming loss of faculty at the University of Zimbabwe College of Health Sciences (UZ-CHS). By 2010, 192 of 314 (61%) faculty positions at UZ-CHS were unfilled. Within the Department of Medicine, 18 of 26 (69%) faculty positions were unfilled. This faculty shortage came at the height of the region’s HIV epidemic and at a time when the total number of medical practitioners in Zimbabwe had decreased 45% over four years (1,818 in 2006 to 998 in 2010) [1]. To meet the health care needs of Zimbabwe, UZ-CHS, the only accredited medical school in the country at that time, sought to develop curricula and programs to enable a small number of faculty to train a large number of doctors highly skilled in HIV care. Previous implementation of problem-based learning at UZ-CHS failed in large part due to increases in the student to faculty ratio at the school [2].

To address the need to provide high quality HIV education with a limited number of teachers, a team-based learning (TBL) teaching method was introduced to the medical school. TBL is a teaching pedagogy designed to maintain the stimulating learning environment of interactive small groups in the face of a high student to faculty ratio [3,4]. In a TBL classroom, the instructor’s duties are changed from being responsible to present all of the course material to preparing and moderating classroom activities while emphasizing key concepts. Students come to class already having read through the assigned material. Individual quizzes are given to assess self-study and acquisition of core concepts. Teams of 4-8 students then apply the knowledge and concepts to challenging problems in an energetic and competitive learning environment. In the U.S., TBL has been successfully introduced into several medical schools with high rates of satisfaction from learners and teachers [5,6].

The ability of TBL to engage a large classroom in the learning of complicated concepts makes it an appealing option to address the current challenges in medical education at UZ-CHS. To our knowledge, descriptions and studies of TBL use in medical schools in Sub-Saharan Africa have not previously been published, despite its potential benefit in low-resource settings. As part of the Novel Education Clinical Trainees and Researchers (NECTAR) program funded by the Medical Education Partnership Initiative (MEPI), a TBL course focused on clinical HIV medicine was designed and implemented for final-year medical students at UZ-CHS. In this article we describe our experience with TBL in a novel setting, a low-income African country, including course design, implementation, impact and sustainability.

## Methods

### Curriculum development and delivery

The development and delivery of the TBL course material was a collaborative effort between faculty at the UZ-CHS and the University of Colorado School of Medicine. A comprehensive competency-based HIV curriculum was created to consolidate the most important concepts and knowledge required to care for HIV-infected patients. The entire course was scheduled in ten 1.5 – 2 hour sessions that took place over the 12 week clinical medicine attachment for final-year medical students. The first course began in August 2011.

All students were provided with the textbook “American Association of HIV Medicine Fundamentals of HIV Medicine” 2007 Edition and a course syllabus describing each session’s learning objectives and assigned reading. The ten sessions covered the following topics: 1. HIV epidemiology, transmission and prevention, 2. Diagnosis and initial evaluation of an HIV patient, 3. Antiretroviral therapy, 4. Treatment failure and drug resistance, 5. Pulmonary complications of HIV, 6. HIV-tuberculosis co-infection, 7. Neurologic complications of HIV, 8. Gastroenterologic complications of HIV, 9. Hematologic and complications and HIV-associated malignancies, and 10. Sexually transmitted infections and dermatologic manifestations of HIV.

For the duration of the course students were divided into ten 5-7 person teams based on their clinical ward assignments. At the beginning of the class session the students were provided with an individual readiness assessment test (IRAT) composed of 4-6 multiple choice questions assessing knowledge from the assigned reading. After IRATs were handed in, the questions were reviewed and the instructor discussed answers with the class until satisfied that the group was prepared to attempt the team exercise. The students then worked together in their teams on 2-3 multiple choice questions based on challenging clinical cases. The cases were based on local experiences and designed for the students to apply their knowledge to complicated scenarios faced frequently in Zimbabwe. Cases were followed by a series of questions with a single best answer. Following completion of the clinical case questions, the teams revealed their answers to the entire class in unison and the instructor facilitated discussion and navigated the class towards the correct answer. Inter-team debate with the use of written material or prior clinical experience was encouraged.

### Curriculum evaluation

A student survey was designed to qualitatively and quantitatively assess the students’ TBL course experience and self-perceived knowledge gained in the course topics. The students were surveyed anonymously at the end of their final session. Students who participated in the course from September – December 2011 and September – December 2012 were surveyed. Nine survey questions assessed the students’ experience with the TBL course format using Likert scale questions. The students were also asked to gauge their knowledge gained for eight of the topics covered during the course (“No improvement”, “Mild improvement”, “Moderate improvement”, “large improvement”, “Massive improvement”). Four open-ended questions asked the students’ opinion on the strengths and weaknesses of TBL, suggestions to improve the course, and what other subjects TBL should be used to teach in their medical curriculum.

## Results

**Figure 1:**
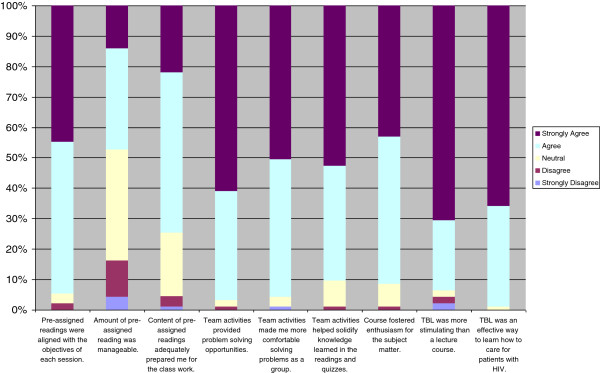
**Student perceptions of team-based learning format.** Percentage of students who strongly agree, agree, are neutral, disagree and strongly disagree with the evaluation statements in the course evaluation.

Ninety-six of 120 students (80%) completed the survey. Ninety-one percent of the students agreed that TBL fostered their enthusiasm for the subject matter and 99% believed it was an effective way to learn to care for patients with HIV (Figure 1). Seventy-five percent of students agreed that the content of pre-assigned readings adequately prepared them for the course. However, 53% of students did not agree that the amount of pre-assigned reading was manageable. Regarding the team activities, the vast majority of students agreed or strongly agreed that they made them more comfortable working in groups (96%) and solidified their knowledge of the course material (91%). Overall, 94% of the students at least agreed and 71% strongly agreed that TBL was more stimulating than a lecture course (Figure 1). Student perceptions on TBL did not significantly differ between the two classes participating in the survey.

**Figure 2:**
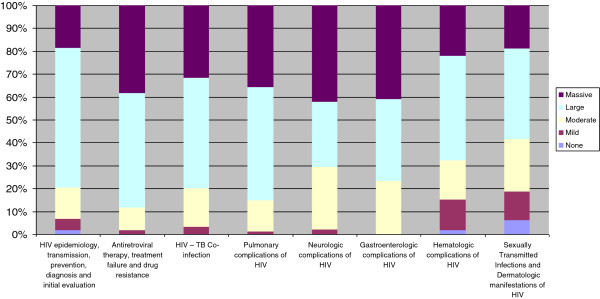
**Students’ self-perceived knowledge gained of selected course topics.** Percentage of students who felt they had massive, large, moderate, mild or no gain in knowledge in each of the content areas covered in the curriculum.

At least 80% of students perceived at least a “moderate improvement” in their knowledge gained in all of the course topics in the survey (Figure 2). Students’ perceived their largest knowledge gained in clinical management of antiretroviral therapy, treatment failure and HIV drug resistance (at least 88% experience large gain). The final medicine examination pass rate of the first group of students exposed to the TBL course was similar to that of previous years that were not exposed to TBL and there was no difference in results on HIV-specific questions. Since the course began in August 2011, seven groups (400 students) have completed the course, which is ongoing. The first two classes were co-administered by a faculty member from the University of Colorado and University of Zimbabwe. Since August 2012, the course has been administered entirely by faculty at the University of Zimbabwe.

The commonest adjectives used by the students when asked about the strengths of TBL were “stimulating” and “interactive”. Several students felt that TBL “made it easier to understand concepts” and “gives room for discussion”. When asked about the weaknesses of the TBL course, the faults identified by the students were the time commitment of the course (“too time consuming”) and amount of required reading (“the reference was too big and too detailed”). Several students also observed that it was “easy for individuals not to participate”. The most common suggestion for the course was that a certificate be awarded upon completion. Eighty percent (77 of 96) of the students surveyed thought that TBL should be extended into other courses in their curriculum, and recommended using TBL to teach other topics of internal medicine and other specialties. Student enthusiasm for the TBL course did not change over time.

## Discussion

The use of TBL for the teaching of an intensive and advanced HIV curriculum to final-year medical students was a success. Despite a limited number of available teachers at the UZ-CHS, our TBL course created a learning environment that was perceived by students as interactive and engaged. Student evaluations suggest that they perceived significant knowledge gains, particularly in advanced and clinically applied topics. The course has been sustained for over two years, has been transitioned to instruction entirely by UZ-CHS faculty, and continues to receive enthusiastic student reviews.

Our positive experience was consistent with previously documented implementations of TBL in medical education at institutions in high-income countries [5,6]. Our experience provides an example that TBL can be successfully implemented in resource-limited teaching settings, which we believe are likely to be most strongly impacted by its use. Price and accessibility of the primary reading resource is an important consideration when planning a TBL course in a resource-limited setting. We benefited from the provision of the course text book (American Association of HIV Medicine Fundamentals of HIV Medicine) through funds from our MEPI program. The cost of appropriate reading material will depend on the subject matter and availability of internet access. Free online resources are also a possible reference source if internet access is universally available to all students, which was not yet the case in our institution.

Our study is limited by its incomplete questionnaire response and reliance on self-reporting of the students’ perception of knowledge gained. Studies have previously shown that students perform equally well or better when taught in a TBL format vs. standard lecture format [7,8]. Our students perceived large knowledge gains in advanced clinical topics. These topics, which tackled challenging concepts through case-based questions, not surprisingly benefited most from the interactive TBL classroom. The final medicine examination pass rate of the first group of students exposed to the TBL course was similar to that of previous years that were not exposed to TBL and there was no difference in results on HIV-specific questions.

A more objective assessment of knowledge gained in our students exposed to TBL will be important to study. However, the evaluation of TBL versus standard lecture format courses can not be completely assessed through examination results because benefits of TBL go beyond transmitting information to students. TBL has been shown to benefit students beyond teaching them the subject matter by improving their communication skills and facilitating their ability to collaborate [9]. Our student and instructors’ observations were consistent with these benefits of TBL.

One drawback of our TBL experience was the time-intensive nature of the course. Many students felt that the TBL course may have taken them away from studying other general medicine topics during their medicine rotation. Our course was intensive in its comprehensive overview of HIV in a setting where the subject matter is of particularly high importance. Despite the demands of the TBL course, the vast majority of students supported the incorporation of TBL into other parts of their medical curriculum. Theoretically, the amount of assigned reading in a TBL course need be no more than a standard lecture course and students should adapt to preparing prior to the course rather than following. Assurance of student preparedness in TBL courses has been previously described as an obstacle for the successful implementation of TBL [10]. Another major obstacle is the need for ongoing faculty development [10]. Our MEPI program has included faculty development, including the implementation of TBL, as a major component of our plans to improve medical education at UZ-CHS. The Departments of Psychiatry, Pediatrics and Physiology are developing similar TBL initiatives.

## Conclusions

We highly recommend considering the institution of TBL into medical curricula in southern Africa. In our experience, TBL will most benefit the learning of challenging clinically-oriented subject matter. TBL is an intriguing and feasible solution to providing high quality medical education at resource-limited institutions with low faculty to student ratios. Further research is needed to assess the impact of TBL on objective learning measures and the feasibility of a more expansive TBL curriculum.

## Abbreviations

HIV: Human immunodeficiency virus; TBL: Team-based learning; NECTAR: Novel Education Clinical Trainees and Researchers; MEPI: Medical Education Partnership Initiative; UZ-CHS: University of Zimbabwe College of Health Sciences; IRAT: Individual readiness assessment test.

## Competing interests

The authors declare that they have no competing interests.

## Authors’ contributions

JG and GF designed and implemented the team-based learning curriculum under the guidance of EA. JG wrote the manuscript with edits provided by GF, EA, TC, and JH. TC and JH, co-primary investigators of the NECTAR MEPI program, identified the need for a revamped HIV curriculum at the University of Zimbabwe College of Health Sciences. All authors read and approved the final manuscript.

## Pre-publication history

The pre-publication history for this paper can be accessed here:

http://www.biomedcentral.com/1472-6920/14/63/prepub
